# In Vitro Incubations Do Not Reflect In Vivo Differences Based on Ranking of Low and High Methane Emitters in Dairy Cows

**DOI:** 10.3390/ani11113112

**Published:** 2021-10-30

**Authors:** Edward H. Cabezas-Garcia, Rebecca Danielsson, Mohammad Ramin, Pekka Huhtanen

**Affiliations:** 1 Department of Agricultural Research for Northern Sweden, Swedish University of Agricultural Sciences (SLU), Skogsmarksgränd, 90183 Umeå, Sweden; Mohammad.Ramin@slu.se (M.R.); pekka.huhtanen@luke.fi (P.H.); 2 Estación IVITA—Maranganí, Universidad Nacional Mayor de San Marcos (UNMSM), Cusco 08258, Peru; 3 Department of Animal Nutrition and Management, Swedish University of Agricultural Sciences (SLU), 75007 Uppsala, Sweden; Rebecca.Danielsson@slu.se; 4 Production Systems, Natural Resources Institute Finland (Luke), 31600 Jokioinen, Finland

**Keywords:** cashew nutshell extract, emitters, GreenFeed, in vitro system, methane production, ranking

## Abstract

**Simple Summary:**

There is a great interest among the scientific community in breeding animals that emit less methane (CH_4_) as a strategy to mitigate the environmental impact of ruminants. The present study ranked individual dairy cows as low and high CH_4_ emitters fed the same diet and evaluated whether the original in vivo ranking was maintained in the in vitro conditions using two contrasting substrates each without or with an antimethanogenic compound. Results do not support a strong effect of rumen microbiome on observed CH_4_ production in vivo, and thus, ranking was not successfully replicated in in vitro conditions. Instead, it appears that animal-related factors such as digesta passage rate are more important drivers of enteric CH_4_ production.

**Abstract:**

This study evaluated if ranking dairy cows as low and high CH_4_ emitters using the GreenFeed system (GF) can be replicated in in vitro conditions using an automated gas system and its possible implications in terms of fermentation balance. Seven pairs of low and high emitters fed the same diet were selected on the basis of residual CH_4_ production, and rumen fluid taken from each pair incubated separately in the in vitro gas production system. In total, seven in vitro incubations were performed with inoculums taken from low and high CH_4_ emitting cows incubated in two substrates differing in forage-to-concentrate proportion, each without or with the addition of cashew nutshell liquid (CNSL) as an inhibitor of CH_4_ production. Except for the aimed differences in CH_4_ production, no statistical differences were detected among groups of low and high emitters either in in vivo animal performance or rumen fermentation profile prior to the in vitro incubations. The effect of in vivo ranking was poorly replicated in in vitro conditions after 48 h of anaerobic fermentation. Instead, the effects of diet and CNSL were more consistent. The inclusion of 50% barley in the diet (SB) increased both asymptotic gas production by 17.3% and predicted in vivo CH_4_ by 26.2%, when compared to 100% grass silage (S) substrate, respectively. The SB diet produced on average more propionate (+28 mmol/mol) and consequently less acetate compared to the S diet. Irrespective of CH_4_ emitter group, CNSL decreased predicted in vivo CH_4_ (26.7 vs. 11.1 mL/ g of dry matter; DM) and stoichiometric CH_4_ (CH_4_VFA; 304 vs. 235 moles/mol VFA), with these being also reflected in decreased total gas production per unit of volatile fatty acids (VFA). Microbial structure was assessed on rumen fluid sampled prior to in vitro incubation, by sequencing of the V4 region of 16S rRNA gene. Principal coordinate analysis (PCoA) on operational taxonomic unit (OTU) did not show any differences between groups. Some differences appeared of relative abundance between groups in some specific OTUs mainly related to *Prevotella.* Genus *Methanobrevibacter* represented 93.7 ± 3.33% of the archaeal sequences. There were no clear differences between groups in relative abundance of *Methanobrevibacter.*

## 1. Introduction

Efficient livestock production will play a key role on achieving sustainability of agricultural systems around the world in such scenario where an increased global demand for food is expected [1,2,3]. Methane (CH_4_) production allows the partial pressure of H_2_ to be low in the rumen, which directs fermentation toward the production of less reduced end-products and more acetate [4]. There are also other possible electron incorporating processes in the rumen such as reductive acetogenesis [5,6] but this process is assumed to be outcompeted by methanogenesis since it is thermodynamically unfavorable [7]. Produced methane represents an energy loss with the extent depending on intake level and diet composition [8]. Due to the negative effect on climate and to reduce the energy loss from the CH_4_ production, a variety of mitigation strategies such as diet manipulation and feed additives have been considered. Methane emissions from ruminants have been shown to be under some genetic control [9,10], and the genetic selection of low-emitting animals has been suggested as one long-term mitigation strategy [11]. 

In addition, previous studies have shown a natural variation between individual animals in CH_4_ yield (CH_4_/DM intake) in the same feeding conditions. The differences in animal physiology and rumen microbiome can contribute to between-animal differences in CH_4_ production. Pinares-Patiño et al. [12,13] found in sheep that fractional passage rate of particulate matter was negatively related to CH_4_ yield. Goopy et al. [14] reported that the higher CH_4_ yield (2.7 g/kg DM intake) in 10 high-emitting ewes was associated with a 5.5 h longer particulate mean retention time in the rumen compared with 10 low-emitting ewes. Goopy et al. [14] also found that low-emitting ewes had a smaller rumen size. Both in animal studies [15] and in model simulations [16], shorter digesta retention time and lower CH_4_ yield were related to reduced diet digestibility. 

Another source of variation may be linked to differences in the structure of rumen microbiome communities, which can be linked to the difference in passage rate. Ruminants seem to have a core microbiome where bacterial domain is dominated by *Prevotella*, *Butyrivibrio*, *Ruminococcus,* unclassified *Lachnospiraceae*, *Ruminococcaceae*, *Bacteriodales*, and *Clostridales* [17]. For the archaeal domain in the cow rumen, *Methanobrevibacter* seems to be the dominant genera [17,18]. Hydrogenotrophic methanogens in rumen are mainly represented by *Methanobrevibacter gottschalkii* clade and *Methanobrevibacter ruminantium* clade, and different CH_4_ production has been identified with the different ratio of these species. *M. gottschalkii* have been related to higher CH_4_ production in ruminants [19,20,21]. A recent study by Greening et al. [22] showed that methanogenesis-related transcript was dominating in high CH_4_ yield sheep, while in low CH_4_-yield sheep, alternative H_2_ pathways were instead upregulated. On the other hand, the low between-cow variability (c.v. = 1.0%) in CH_4_ per unit of volatile fatty acids (CH_4_VFA) [23] does not support large differences in rumen fermentation pattern [24].

The main objective of the present study is to elucidate the role of specific ruminal factors (i.e., fermentation profile, bacterial, and archaeal populations) contributing to the observed between-cow differences in in vivo CH_4_ production as measured by an open-circuit head chamber system. Our hypothesis was that by using rumen inoculum from low-emitting cows, in vitro CH_4_ production should be reduced compared with rumen fluid from high-emitting cows. Two different diets (forage-alone and mixed) were used to investigate possible interactions between inoculum and substrate. An antimethanogenic substance known as cashew nutshell liquid (CNSL), which has shown a clear reduction in our previous in vitro studies [25], was used to investigate possible interactions between rumen inoculum and additive. Finally, by establishing relationships between the rumen microbiome (i.e., especially methanogenic population) and the host animal, a better understanding of the contribution of both sources on the observed variation in in vivo CH_4_ production could be achieved. 

## 2. Materials and Methods

Seven in vitro incubations were performed to evaluate the potential of an in vitro technique on replicating a previous animal ranking according to residual CH_4_ production in vivo and its implications in terms of fermentation balance and rumen microbiome.

### 2.1. Animal Data

All procedures involving animals were approved by the Regional Ethics Committee of the Swedish University of Agricultural Sciences in Umeå, Sweden. In vitro incubations were performed in connection with two feeding experiments conducted with intact Nordic Red dairy cows in 2013 and 2014, respectively, at the Röbäcksdalen Research Centre. The first four in vitro incubations were conducted in parallel with rumen fluid collection from individual cows taken concurrently to the in vivo trials as part of the larger study conducted by the RuminOmics EU collaborative project [26] at four different time points. The last three in vitro incubations were performed at the end of the study of Pang et al. [27], at three different time points. All cows were fed *ad libitum* a total-mixed ration (TMR) delivered into automatized feed bins (Insentec Ltd., Lemelerveld, The Netherlands). The basal diet consisted of primary growth timothy grass silage (*Phleum pratense*) and cereal grain concentrates (600/400 g/kg on a DM basis) and was rather similar across the studies. The average chemical composition of grass silages was: DM= 301 ± 30.1 g/kg; OM = 927 ± 14.6 g/kg DM; CP = 162 ± 16.4 g/kg DM; and NDF = 566 ± 101 g/kg DM. The chemical composition of TMR diets offered to the cows was: 383 ± 25.8 g/kg of DM, 154 ± 13.3 g/kg CP, and 433 ± 54.6 g/kg NDF. Body weight was recorded after morning milking for three days before the feeding experiments and on the last three days of each experimental period, and therefore, mean BW values were obtained for each cow. Donor cows were on average 620 ± 84.3 kg.

### 2.2. In Vivo CH_4_ Production and Animal Ranking for Low and High Emitters

Gas production (CH_4_ and CO_2_) were recorded daily by two transportable open-circuit head chamber systems attached to concentrate feeding stations (GF; GreenFeed system, C-Lock Inc., Rapid City, SD, USA) as described by Huhtanen et al. [28] and Hristov et al. [29]. Span gas calibrations (N_2_ and a mixture of CH_4_ and CO_2_) were performed once a week, and CO_2_ recovery tests were conducted every second week during each experimental period. In both studies [26,27], the GF system was programmed to allow each animal to visit the two GF units at 5 h intervals over the day. During each visit, the cows were given 8 servings of 50 g of a commercial concentrate (Solid 220, Lantmännen, Malmö, Sweden) at 40 s intervals during each visit. No differences were detected between low and high emitters on concentrate consumption from the GF units. 

In total, gas fluxes by the GF equipment were measured on 100 and 32 individual cows for the feeding experiments conducted in 2013 and 2014, respectively. Although the two GF units were operated continuously, gas production data within the last two weeks of each experimental period were used for ranking of cows according to their CH_4_ production. The cows were ranked as low and high emitters based on the residual CH_4_ production calculated as a difference between observed and predicted CH_4_ production. 

Predicted CH_4_ production was estimated by accounting the fixed effects of DMI (x_1_), BW (x_2_), and experimental period (x_3_) by linear regression and obtained values expressed as least square means.

Predicted CH_4_ = DMI + BW + Period

Period was included in the model to remove variation resulting from possible differences in diet composition between periods. Once animal ranking was established for each in vivo study, seven low and seven high emitters were selected as donor animals and grouped in seven pairs (one low and one high) for collection of rumen fluid (four pairs of cows taken from the study by Wallace et al. [26] and the last three pair of cows taken from the study conducted by Pang [27]). A sample of rumen fluid was taken from each pair of donor cows by stomach tube two hours after morning feeding. The first liter of rumen fluid was discarded to avoid saliva contamination, and the next 0.5 L was collected directly into prewarmed thermos flasks previously flushed with CO_2_. Stomach tube device was cleaned thoroughly with tap water before collection of rumen fluid from the second cow within pair. Rumen fluid was transported to the laboratory within 15 min prior to the in vitro incubations.

### 2.3. In Vitro Incubations and Laboratory Procedures

Samples of timothy grass silage and barley were dried in a forced air oven at 60 °C for 48 h and milled through a 1 mm screen using a Retsch SM 2000 cutting mill (Retsch GmbH, Haan, Germany), and ground samples were then stored in sealed glass jars until required. The chemical composition of the feeds used as substrates in the in vitro incubations is shown in Table 1.

Two substrates were used in the in vitro incubations: 100% grass silage (S) and a mixture of 50% grass silage and 50% barley (SB), each without or with CNSL as a CH_4_ inhibitor. For the S diet, 1000 mg of grass silage was weighted into serum bottles (250 mL; Schott AG, Mainz, Germany), whereas a mixture of 500 mg of grass silage and 500 mg of barley was weighted for the SB treatments, respectively. Cashew nutshell extract was prepared according to the extraction procedure by Philip al. [30]. For the CNSL treatments, 10 μL of cashew nut shell extract was dissolved in 490 μL of ethanol (99.5%), and this liquid (CNSL) was then transferred into each serum bottle. The ethanol of CNSL treatments was evaporated by leaving the serum bottles at room temperature overnight. 

The next day, the in vitro incubation took place using rumen fluid obtained from one pair of donor cows previously ranked as low and high emitters. The pH of rumen fluid of both cows was recorded prior to the in vitro incubation. After swirling, four 1 mL subsamples were pipetted into freeze-resistant tubes (2 mL capacity), immediately frozen in dry ice, and stored at −80 °C for further analysis of microbial community structure. Two extra aliquots of 3 mL each were pipetted into centrifuge tubes with 0.6 mL of 25% metaphosphoric acid (5:1 rumen fluid: acid ratio) and stored at −20 °C for the analysis of volatile fatty acids (VFA). Rumen fluid was filtered through four layers of cheesecloth and then mixed with buffered mineral solution [31] (20:80 *v/v*) supplemented with peptone (Merck, Darmstadt, Germany) at 39 °C with constant stirring and continuous flushing with CO_2_. Finally, the serum bottles were filled with 60 mL buffered rumen fluid and placed in a water bath at 39 °C for 48 h. All treatments were performed in triplicate with three blanks included for each inoculum. The order of starting incubations with low- and high-emitter rumen fluid was switched between incubation runs.

Gas production was measured using a fully automated system (Gas Production Recorder, GPR-2, Version 1.0 2015, Wageningen, UR), with readings made every 12 min and corrected to the normal air pressure (101.3 kPa) [32]. Measurement of CH_4_ in vitro were performed according to Ramin and Huhtanen [33] on gas samples (0.2 mL) collected from the headspace of each bottle with a gas tight syringe (Hamilton, Bonaduz, Switzerland) during incubation at different time points: 2, 4, 8, 24, 32, and 48 h. Concentration of CH_4_ was determined with a Varian Star 3400 CX gas chromatograph (Varian Analytical Instruments, Walnut Creek, CA, USA) equipped with a thermal conductivity detector. Calibration gas was completed using a standard mixture of CH_4_ and CO_2_ (100 mmol/mol) prepared by AGA Gas (AGA Gas AB, Sundbyberg, Sweden). Peaks were identified by comparison with the standard gas. A logarithmic model of incubation time (h) vs. CH_4_ concentration (%) was developed for each bottle to estimate CH_4_ concentration at time intervals of 0.2 h (the gas system recorded total gas production every 0.2 h). Methane production was estimated for each 0.2 h interval as described by Ramin and Huhtanen [33] and corrected for blanks. The two-pool Gompertz model [34] was fitted to the data by the NLIN procedure of SAS (SAS Inst. Inc., Cary, NC, USA). The resulting estimated kinetic parameters were used as input to run a mechanistic rumen model with a 50 h rumen retention time (20 and 30 h in rumen nonescapable and escapable pools) to predict the in vivo CH_4_ production at maintenance level of intake. Details of the calculations are described by Ramin and Huhtanen [33].

At the end of 48 h in vitro incubation, the pH was measured. Fluid samples (1 mL) were taken from each bottle (replicate) and two pooled samples (3 mL) obtained for each treatment and processed for VFA analysis as described before. The VFA concentrations were determined by gas chromatography using the method of Playne [35]. The VFA ratios acetate/propionate and propionate/butyrate were calculated, and the lipogenic: glucogenic ratio of VFA was determined as (acetate + butyrate)/propionate. Production of CH_4_ per mole of VFA (CH_4_VFA) was calculated based on VFA stoichiometry Equations [23]:

CH_4_VFA (mmol/mol of VFA) = 0.5 × C_2_ − 0.25 × C_3_ + 0.5 × C_4_

where C_2_, C_3_, and C_4_ are molar proportions (mmol/mol) of acetate, propionate, and butyrate, respectively, of the sum of these VFA. 

### 2.4. Analyses of Rumen Microbiome

#### 2.4.1. DNA Extraction

The DNA was extracted from rumen fluid samples in triplicate using 300 μL sample per replicate and the FastDNA^®^ Spin kit (MP Biomedicals, LLC, Solon, OH, USA). The extraction step was performed in accordance with the manufacturer’s protocol except for an additional purification step to remove PCR-inhibiting component as suggested by the manufacturer. In brief, samples were washed and resuspended with a humic acid wash solution, which contained sodium phosphate buffer, MT buffer (provided with the kit), and 5.5 M guanidine thiocyanate. The samples were transferred to SPIN filter, following settling of the binding matrix. In the final step, DNA was eluted by adding 50 μL DNase/pyrogen-free water (provided with the kit). The DNA concentration was quantified using a Qubit fluorometer (Life Technologies, Carlsbad, CA, USA), with a range between 5.2–56 ng/µL.

The 16S rRNA amplicon libraries were constructed with a two-step PCR. The first PCR simultaneously targeted the V4 region of both bacteria and archaea, using the primers 515´F (GTGBCAGCMGCCGCGGTAA) and 805R (GGACTACHVGGGTWTCTAAT) [36]. The reaction mixtures were set up using Phusion high-fidelity DNA polymerase (Thermo Fischer Scientific, Hudson, NH, USA). The reaction mixture contained 5 µL Phusion buffer, 0.5 µL (10 mM) dNTP, 0.75 µL DMSO, and 0.25 µL (2 U/µL) Phusion polymerase. The first PCR reaction contained 0.5 µL (10 µM) of each primer, Phusion mix, and DNA template. Amplification was performed under the following conditions: initial denaturing step at 98 °C for 30 s, 20 cycles of: 10 s at 98 °C, 30 s at 60 °C, 4 s at 72 °C, and a final extension at 72 °C for 2 min. The PCR products were checked for size and quality by electrophoresis. Samples were then purified using Agencourt AMPure XP (Becker Coulter, Brea, CA, USA), using a magnetic particle/DNA volume ratio of 0.8:1. The second PCR reaction contained 10 µL purified DNA product, Phusion reaction mix and 1 µL each of the primers 5’-AATGATACGGCGACCACCAGATCTACACX_8_ACACTCTTTCCCTACACGACG-3 and 5’-CAAGCAGAAGACGGCATACGAGATX_8_GTGACTGGAGTTCAGACGTGTGCTCTTCCGATCT-3’, where X_8_ in the primer sequence, represented a specific Illumina-compatible barcode. Detailed information about these primers can be found in Hugerth et al. [36]. The barcodes (Eurofins Genomics) were combined, giving a unique combination of barcodes for each sample and thereby allowing for multiplex analysis in the sequencing. The following conditions were used for the second PCR step: initial denaturing at 98 °C for 30 s, 8 cycles of 10 s at 98 °C, 30 s at 62 °C, 5 s at 72 °C, and a final extension at 72°C for 2 min. The PCR products were checked by electrophoresis and purified using Agencourt AMPure XP. Each sample was then diluted to the same DNA concentration of 20 nM and pooled to one sample library. The pooled library was sequenced on the MiSeq system (Illumina, Inc., San Diego, CA, USA) at Science for Life Laboratory/NGI (Solna, Sweden).

#### 2.4.2. 16S rRNA Data Analysis

Analysis of 16S sequencing data was performed using the Nextflow computational pipeline ampliseq v1.1.2 (https://github.com/nf-core/ampliseq, accessed on 21 September 2020). In brief, raw sequencing reads were quality checked initially using FastQC [37], followed by trimming of adaptor sequences from the reads using cutadapt v2.7 [38]. Quality distribution of trimmed reads was then analyzed using tools provided in QIIME2 software package v2019.10 [39]. Demultiplexed sequences were quality-filtered and trimmed, denoised, dereplicated, and filtered for chimeric sequences using pair-ended DADA2 [40], resulting in exact amplicon sequence variants (ASVs) tables. The ASVs were taxonomically classified from phylum to species level clustered with 99% similarity using the SILVA v132 database [41] by applying Naive Bayes classifier implemented in QIIME 2 [39], trained on the preprocessed database. Following taxonomic classification of ASVs to OTUs (operational taxonomic units), the OTUs classified as Mitochondria or Chloroplast were removed. The final OTU table was filtered based on the criteria that the OTU comprising ≥30 reads (approx. abundance of >0.0001% in the samples altogether) in at least three samples were retained. QIIME 2 was used to assess alpha-diversity through Pielou’s Evenness, Shannon, and Faith’s phylogenetic diversity metrics. Beta-diversity was estimated using Bray–Curtis dissimilarity, Jaccard index, weighted and unweighted UniFrac distance, also implemented in QIIME2. Archaeal sequences were filtered out separately in a second step, and relative abundance was calculated in relation to total archaeal counts. Basic local alignment search tool (BLAST) against the rRNA/ITS database was used to further classify methanogens representative OTU sequences of interest [42]. Raw reads have been deposited in European Nucleotide Archive (ENA) with accession number PRJEB48001. 

### 2.5. Statistical Analysis

Differences in animal performance and gas production data in vivo as well as the concentrations and proportions of VFA prior to the in vitro incubations were compared between groups of low and high emitters and including the effect of pair of cows in the model as a covariate by using the PROC GLM of SAS (version 9.4; SAS Institute Inc.). Data for in vitro measurements (total gas, predicted CH_4_ production, VFA production) were analyzed using the PROC MIXED of SAS. The statistical model used for the analysis was:

Y*_ijkl_* = μ + E*_i_* + D*_j_* + C*_k_* + E×D*_ij_* + E×C*_ik_* + D × C*_jk_* + E × D × C*_ijk_* + R_l_ + e*_ijkl_*

where, E, D, and C are the emitter (low vs. high), diet (S vs. SB), and CNSL (without vs. with), respectively, including their respective interactions. The effect of in vitro incubation, run (R) was considered as random, and e*_ijkl_*~N (0, σ^2^e) is the random residual error. Least square means are reported, and mean separation was performed by least significant difference to test differences between treatments.

Principal coordinate analysis (PCoA) was performed to identify possible clustering patterns among the samples. The PCoA was based on Bray–Curtis distance metrics and analyzed using the PAST software (http://folk.uio.no/ohammer/past/, accessed on 3 November 2020) [43]. The validity of clustering patterns was confirmed by a distance-based nonparametric MANOVA (Bray–Curtis distance with 9999 permutations, PAST software). For evaluating effects of differences in OTUs/sequences between clusters (Y*ij*, *n* = 14), the following MIXED model was used:

Y*ij* = E*_i_* + R*_j_*+ e*_ij_*

where E is the fixed effect of emitters (low and high; *_i_* = 2), R refers to effect of in vitro incubation run (*n* = 7), and e*_ij_* is random error. All differences were declared significant at *p* < 0.05.

## 3. Results

### 3.1. In Vivo Measurements

Animal performance and gas production on-farm conditions for both emitter groups are presented in Table 2. Although high emitters were slightly heavier and more efficient than the low emitters, no statistical differences were detected among groups in these variables (*p* > 0.05). The total enteric CH_4_ production of the low emitters represented 75% of the observed emissions for the high emitters across pairs of cows. The selection of both low and high emitters on the basis of residual CH_4_ production was successful for obtaining the aimed two contrasting groups of cows (*p* < 0.01), and this was also reflected in a greater CH_4_ yield (+5.2 g/kg DMI) in the high emitters (*p* = 0.02). Concentrations and proportions of VFA collected from rumen fluid of donor cows prior to the in vitro incubations are presented in Table 3. No statistical differences were detected between low and high emitters in the VFA parameters (*p* > 0.05).

### 3.2. Total Gas and CH_4_ Production In Vitro

The results for total gas production and predicted CH_4_ production are presented in Table 4. Generally, the effect of the emitter was by far less consistent compared to both diet and CNSL, and it was poorly replicated in in vitro conditions. This effect was only significant for the rate of predicted in vivo CH_4_ production (*p* ≤ 0.05). Conversely, the diet effect was significant (*p* < 0.01), indicating that more gas and CH_4_ were produced from SB compared to the S diet.

The rates of total gas production and predicted in vivo CH_4_ were also in agreement with increased gas production observed in the SB diet. The diets did not differ in the proportion of CH_4_ over the total gas production (*p* = 0.52). The addition of CNSL consistently decreased (42%) both the total gas and CH_4_ production (*p* < 0.01). This trend encompassed the rate of total gas production, but it was opposite for the rate of predicted CH_4_ production (*p* < 0.01). A significant interaction between emitter and diet was only found in total gas production per mmol of VFA (*p* < 0.01). Despite emitter group, the addition of CNSL increased the rate of predicted in vivo CH_4_ production and CH_4_VFA (*p* ≤ 0.03), and this was reflected in the total gas production per unit of VFA (*p* < 0.01; data not shown). 

### 3.3. Rumen Fermentation

The concentrations and proportions of VFA, as well as stoichiometry CH_4_ after 48 h of in vitro incubation are presented in Table 5. Both total VFA production (corrected for blank), and VFA concentrations were not (*p* ≥ 0.11) different between the two sources of inoculum. The proportion of propionate was lower for the low compared to the high emitters (280 vs. 301 mmol/mol VFA, respectively). A reverse trend (*p* ≥ 0.06) was found for acetate which was reflected in a statistical difference for molar proportions (600 vs. 584 for the low and high emitters, respectively). The SB diet increased on average molar proportion of propionate (+28 mmol/mol) and consequently less acetate compared to S diet (*p* < 0.01). The addition of CNSL consistently increased propionate and decreased acetate (*p* < 0.01), and this showed a reverse trend related to total VFA profiles (*p* < 0.01). Significant interactions were detected between inoculum sources with both diet and CNSL effects on total VFA profiles (*p* ≤ 0.01). The additive increased propionate more in high emitters compared with low emitters. The addition of CNSL in combination with diet further decreased CH_4_ production in vitro (*p* < 0.01). Both CH_4_VFA and CO_2_VFA differed among CH_4_ emitters, diet, and CNSL (*p* < 0.01). The source of inoculum did affect the final pH of buffered rumen fluid after 48 h of incubation (*p* = 0.02).

### 3.4. Analysis of Microbial Composition

The structure of the rumen archaeal and bacterial community in the dairy cows was characterized by sequencing the V4 region of 16S rRNA gene with Illumina MiSeq. After trimming and quality check, in total, 4,856,845 sequences were obtained from 14 samples with an average of 346,918 sequences per sample (range 95,581–545,789, median 344,136). The threshold level for OTU abundance was set to >0.001%. The number of archaea sequences was 40,622, which represented 0.8% of total sequences, with an average of 2901 sequences per sample (range 328–5554, median 2929).

#### 3.4.1. Bacteria

**Figure 1:**
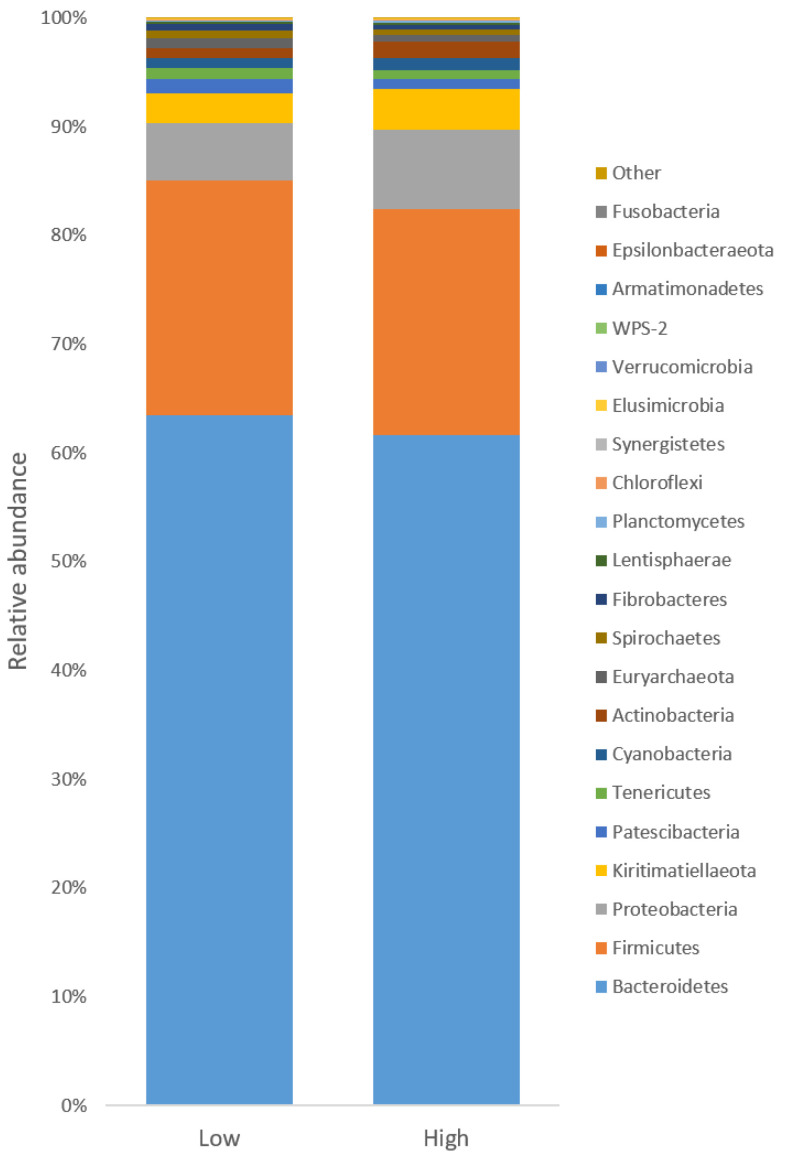
Relative abundance of all sequences at phyla level in low and high emitters.

The bacterial population was represented by 21 different phyla, with 16 phyla found across all samples. The bacterial composition was dominated by the Bacteroidetes phylum, representing on average 62.5 ± 4.59%, average values ± s.e., of all sequences, followed by Firmicutes (21.2 ± 3.39%) and Proteobacteria (6.2 ± 4.42%). The remaining phyla represented less than 10% of all sequences (Figure 1). At the genus level, *Prevotella* dominated, representing on average 35.6 ± 3.73% of all sequences. Other abundant genera were *Ruminococcus* (2.2 ± 0.63%) and *Succiniclasticum* (1.3 ± 0.55%), as well as unclassified *Prevotellaceae* (6.5 ± 0.32%) unclassified *Succinivibrionaceae* (5.2 ± 0.41%), unclassified *Rikenellaceae* (4.9 ± 0.56%), and unclassified *Ruminococcaceae* (3.0 ± 0.26%).

#### 3.4.2. Archaea

The archaeal community was only represented by the phyla Euryarchaeota. Orders were represented by Methanobacteriales (96.8 ± 0.27%) and Methanomassiliicoccales (2.4 ± 2.47%). Euryarchaeota was dominated by the genus *Methanobrevibacter*, which represented 93.7 ± 3.33% of the archaeal sequences, followed by *Methanosphaera* and unclassified members of the family Methanomethylophilaceae, representing 3.2 ± 2.04% and 2.4 ± 2.47% of the archaeal population, respectively. After using the BLAST tool against the rRNA/ITS database for all the archaeal OTUs, it was possible to identify sequences at the species level. *Methanobrevibacter* were mainly represented by species belonging to the two main groups RO (*M. ruminantium* and *M. olleyae*) (21.5 ± 8.43%) and SGMT group (*M. smithii*, *M. gottschalkii*, *M. millerae*, and *M. thaueri*) (69.8 ± 10.73%).

### 3.5. Differences in Microbial Community Structure between Low and High Emitters

**Figure 2:**
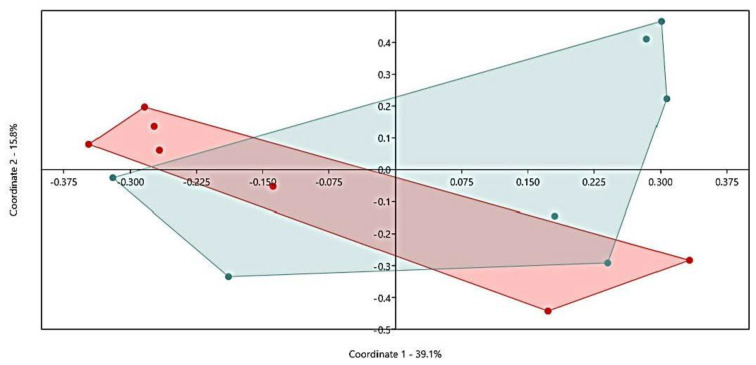
Principal coordinate analysis (PCoA) defining the relationship between samples based on the bacteria operational taxonomic unit (OTU) level. Colors represent different CH_4_ groups: green= low emitters, and red = high emitters. Axes describe percentage of variance.

**Figure 3:**
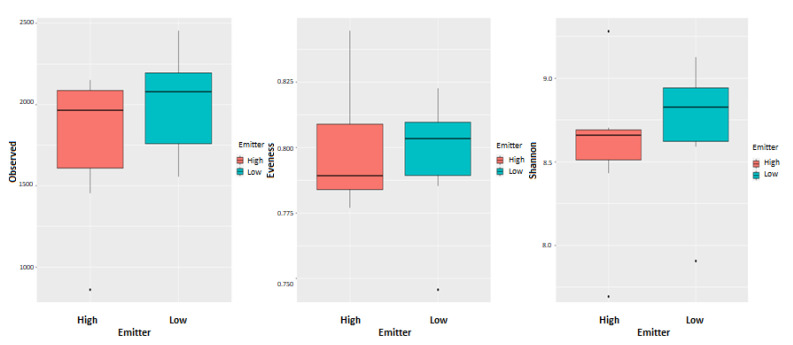
Alpha diversity analysis. Boxplots representing variations in alpha diversity in the rumen fluid: high (red) and low (green) emitters respectively. Alpha diversity metrics include observed OTUs, Evenness, and Shannon indexes are for both bacteria and archaea. High *n* = 7, Low *n* = 7.

**Figure 4:**
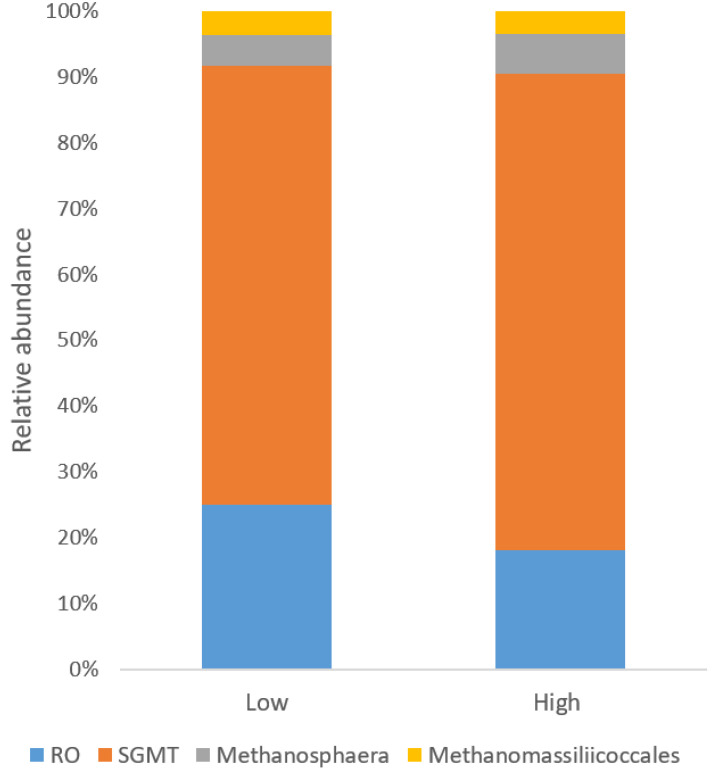
Relative abundance of archaea sequences in low and high emitters. RO is represented by *Methanobrevibacter* species *of Ruminantium* and *Olleyae* with high similarity (>97%), and SGMT is represented by *Methanobrevibacter* species *Smithii, Gottschalkii, Millerae* and *Thaueri* with high similarity (>97%). Sequences within family *Methanosphaera* and order *Methanomassiliicoccales* are also represented.

There was no clear difference (*p* = 0.173) in the community structure between low and high emitters, which is shown in the PCoA plot on all OTUs (Figure 2). Alpha diversity indicators; Shannon, Evenness, and observed OTUs were not different (*p* = 0.482, 0.749, and 0.277, respectively) between low and high emitters (Figure 3). Comparisons of relative abundance between groups showed only a difference in Chloroflexi that was two-fold higher in high CH_4_ yielding group, no other Phyla differed. At the genus level, no difference was found. At OTU level, there were differences in some specific OTUs mainly related to *Prevotella spp*. For archaea, the relative abundance was on average 0.9 ± 0.51%, and 0.7 ± 0.28% for low and high emitters, respectively. Archaea sequences were further filtered out separately, and at a species level, the two clades within *Methanobrevibacter* were compared according to analysis suggested from previous studies. In the present study, there were no difference in the groups, RO group (*p* = 0.272) low group (25.0 compared to high 18.1, SEM = 4.01) for SGMT group (*p* = 0.484) (66.8 compared to high 72.4, SEM = 5.32), and the total relative abundances for archaea sequences in the different groups can be found in Figure 4.

## 4. Discussion

To the best of our knowledge, the present study was the first attempting to demonstrate if the differences between animals in vivo CH_4_ emissions are due to rumen microbiome by using rumen fluid from low- and high-emitting cows as inoculum in in vitro conditions. By using an in vitro system, animal-related factors such as rumen volume and passage rate are excluded. An earlier study by Wang et al. [44], evaluated the potential usefulness of rumen fluid collected from slaughtered cattle to detect differences in CH_4_ emission of cattle types by an in vitro test and concluded that the in vitro test is not sensitive enough to be used as a tool to follow genotypic differences in CH_4_ yield, especially at individual-animal level. In the present study, no differences were found in the gas production in vitro between low and high emitters, and this was aligned with similar microbial communities among groups. 

### 4.1. GreenFeed vs. In Vitro Gas Measurements

In vitro techniques have been traditionally used for screening diets and evaluation of feed additives on enteric CH_4_ production upon collection of rumen fluid from donor animals [20,33], thus allowing the incubation of large numbers of samples that can be analyzed at the same time at a lower cost when compared to in vivo measurements. The in vitro gas production system used in the present study as described by Ramin and Huhtanen [33] can predict in vivo CH_4_ production by using the rumen kinetic model after running the system for 48 h in a rumen model. In the same study, predicted in vivo CH_4_ emission decreased from 7.8 to 6.0% of total GE intake with an increased amount of substrate in their system. Similar values have been reported for dairy cows at the maintenance and production level of intake [45]. Ramin and Huhtanen [33] also found that there was a strong relationship between predicted CH_4_ production using the VFA stoichiometric equations (CH_4_VFA) and measured CH_4_ using their in vitro system (R^2^ = 0.97). In an extensive study by Danielsson et al. [20] using the same in vitro system as used in the present study, a good relationship between in vitro predicted and observed CH_4_ production was observed for 49 different diets in which the respiration chamber was used for measuring CH_4_ production.

The GF system has been validated both directly and indirectly against respiration chamber data. In a recent study, Huhtanen et al. [46] showed that CH_4_ production measured by the GF performed well compared to values predicted by empirical models derived from large respiration chamber data. This indirectly suggests that enteric CH_4_ production can be reliably measured by the GF system. Conversely, in a direct comparison using 20 studies, Hristov et al. [47] found a good relationship between CH_4_ production measured by respiration chambers and the GF method. Cabezas-Garcia [48] collected data from 10 in vivo studies in which the GF was used to measure CH_4_ production. Between-cow variation was higher than the residual variation, demonstrating high repeatability (0.69) of the GF technique in measuring CH_4_ production in dairy cows.

### 4.2. Rumen Microbiome and CH_4_ Production

#### 4.2.1. Bacteria

No significant difference was observed for the bacterial community structures between low and high emitters. At phyla level, differences were only present in Chloroflexi. Several OTUs that differed in relative abundance between clusters were classified to *Prevotella*, which is usually the main bacterial genus represented in the cow rumen, with many different species observed [49,50]. Comparison at genus level of *Prevotella* did not reveal any differences between groups of low or high emitters. There was a difference between groups at OTU level, i.e., several OTUs of *Prevotella spp*. had a higher abundance in high group compared to low and several other OTUs had higher relative abundance in high compared to low emitters.

It is known to be a great variation in the ability of different *Prevotella* species to utilize certain substrates, a nutritional adaptation as an advantage in the rumen environment with different components available through carbohydrate and protein feeds given to the cow [51,52]. On the other hand, this versatility of substrates makes the role of the *Prevotella* even harder to understand [21]. The potential role of *Prevotella* is difficult to explain in any case, as a large proportion of the population is represented by uncultured species [49]. Further investigation of the phenotypes of these dominant ruminal bacteria is needed to better understand its role and relation to animal and dietary factors.

#### 4.2.2. Archaea

For the total abundance of archaea, no significant difference was observed between the different groups. At the species level, where the relative abundance of *M. ruminantium* clade was not different but had a numerical higher abundance in the low emitters, the *M. gottschalkii* clade had a numerical higher abundance in the high CH_4_ emitter group. This association between the two groups of *Methanobrevibacter* species, and CH_4_ production agrees with previous findings [19,20]. A feature in common and which is specific for all methanogens is the use of methyl coenzyme M-reductase (Mcr) [53]. In the last step in methanogenesis, the methyl group in methyl coenzyme M is reduced to CH_4_ by Mcr, and coenzyme M is regenerated. Hydrogenotrophic methanogens in rumen are mainly represented by *Methanobrevibacetr. M. gottschalkii* has the capacity to express both Mcr I or Mcr II at low and high H_2_ pressure, while *M. ruminatium* seems to express only methyl coenzyme Mcr I, which is used at lower H_2_ pressures [18]. The level of H_2_ in the rumen might differ between the groups due to animal factors such as passage rate.

#### 4.2.3. Alternative H^+^ Sinks

What is known is that there are other possible electron incorporating processes: for example, hydrogenotrophic bacteria in the rumen, such as acetogens, can reduce CO_2_ to form acetate by the reductive Acetyl-CoA or Wood–Ljungdahl pathway (reductive acetogenesis) [54,55]. In the typical ruminal fermentation, methanogens can outcompete acetogens by using H_2_ at low level. The process is thus believed not to occur to any significant extent [56,57]. In our studies, no differences were found between high and low emitters in acetogens, at genus level, such as *Eubacterium, Blautia, Acetitomaculum*, or *Oxobacter.* Anyhow, in a study by Greening et al. [22], the hydrogen production and consumption pathways related to CH_4_ production were investigated by metatranscriptomic analysis. It was found that the methanogenesis-related transcript was dominating in high CH_4_-yield sheep, while in low CH_4_-yield sheep, alternative H_2_ pathways were instead upregulated. 

### 4.3. Animal-Related Factors

In the present study, no differences were found neither in terms of total VFA production nor molar proportions between rumen fluid collected from low and high emitters fed the same basal diet prior to the in vitro incubations. In addition, to diet composition, enteric CH_4_ production is largely driven by a number of control mechanisms that the host animal exerts on its own gut microbiota which in turn is reflected their diversity, size, and activity on the fermentation substrate. Among animal-related factors, saliva production, rumen volume, and passage rate (which is directly related to intake) are particularly important since these physiological mechanisms influence on the physical structure and dynamics of gut digesta that may differ among individual animals [12,14,58].

Lower CH_4_ yield has been reported in sheep with smaller rumen volume and short mean retention time [12,14], and these observations in vivo are consistent with results found in the modeling study by Huhtanen et al. [16]. Thus, it is expected that animals with a higher reticulorumen volume exhibit increased retention time of rumen digesta and consequently greater amounts of fermented feed than smaller animals when consuming similar amounts of a common diet, with this resulting in higher CH_4_ production per unit of intake [12,14,59]. In smaller animals less substrate is available for methanogenesis.

Because CH_4_ is produced from fermentable substrate, it is expected that CH_4_ production decreases with reduced digestibility, which is in turn associated with faster passage rate. Løvendahl et al. [15] reviewed data from several studies and showed a positive relationship between CH_4_ yield and digestibility. Ørskov et al. [60] showed that diet digestibility and digesta passage rate were strongly and negatively correlated. Similar associations between passage rate, digestibility, and CH_4_ production were observed by a mechanistic modeling approach [16]. Reduced digestibility cannot explain lower CH_4_ yield with increased DMI, since diet digestibility has been shown to be lower in low emitters than in high emitters, and a small rumen volume (likely associated to low-emitter animals) could also limit intake potential of forages. Therefore, selecting low-emitting animals may compromise ruminants’ unique ability of transforming roughages into human food (e.g., milk and meat). 

Although no study assessing the relationship between passage rate and fermentation patterns was found, experimental evidence has shown that an increased feeding level leads to changes in the rumen fermentation with increased propionate concentrations [61,62]. Jonker et al. [63] found that the ruminal fermentation pattern was significantly related to CH_4_ yield with the ratio of (acetate + butyrate) / (propionate + valerate) and the propionate concentration alone being the best single predictor of CH_4_ yield. The level of H_2_ in the rumen is of critical importance, as it regulates the upstream oxidations in the glycolysis and also the level of the fermentation products (e.g., acetate and butyrate). Hydrogen is mainly produced via the action of hydrogenases, transferring electrons to H^+^ while reoxidizing NADH to NAD [23,64]. This reaction requires low levels of hydrogen to proceed, and in case the consumption of hydrogen by the methanogens is inefficient, electrons are instead to a higher degree transferred to acetyl-coA to form butyrate. As an alternative, the electron from the reoxidation of NADH can also be incorporated in propionate, via production of succinate or lactate. In this case, less H_2_ can be used by methanogens, and less CH_4_ is formed. In studies by Wang et al. [65,66], the concentration of dissolved H_2_ in the rumen was negatively correlated with acetate and positively with propionate molar percentages, but an association with propionate molar percentage was not observed in an earlier study by Wang et al. [67]. The higher proportion of propionate with the SB diet was reflected in reduced CH_4_ production. Increased propionate reflected on increased supply of fermentable substrate as the greater total gas production indicate. Increased passage rate may lead to changes in the proportions of substrates fermented more likely affecting the NDF pool than the neutral detergent soluble fraction (NDS). As available substrate changes, this may also contribute to differences in microbial communities.

At present, it is unknown if between-cow differences in passage rate have the same effect as the observed for feeding level. Results from the meta-analysis by Cabezas-Garcia et al. [24] do not support a strong relationship between passage rate and VFA profile, and only small differences in VFA proportions were found. In line with this, Pinares-Patiño et al. [12] found much greater variability in passage rate than in rumen fermentation patterns, despite the quite large differences in CH_4_ production in individual sheep. Cabezas-Garcia [48] demonstrated from the primary data of Kittelmann et al. [68] that rumen fermentation pattern (CH_4_VFA) explained a relatively small proportion of the variation in CH_4_ yield (R^2^ = 0.16). It was unclear if the observed variability was either associated to microbiome, passage rate, or a combination of both effects. Shi et al. [19] suggested that differences in microbiome communities affected passage rate. However, it could also be that differences in passage rate and rumen volume affect rumen microbiome. 

Both reduced digestibility and improved efficiency of microbial protein synthesis as suggested by a number of studies [62,69,70] appear to be the main factors contributing to reduced CH_4_ yield with increased passage rate (and feeding level). As more energy sources are diverted to microbial growth with the increased efficiency of microbial cell synthesis, more fermented carbon is partitioned to microbial cells instead of VFA and fermentation gases [71,72]. In addition, microbial cells are more reduced than fermented carbohydrates [73,74] and can act as an effective H_2_ sink. Therefore, the increased efficiency of microbial growth promotes lower CH_4_ production.

## 5. Conclusions

The results of the present study suggest that between-cow differences in CH_4_ production (low and high emitters) are more likely related to physiological differences between animals (e.g., rumen volume and passage rate). Indeed, both rumen fermentation and microbiome data support these findings. It is important to note that the reduced CH_4_ production with increased passage rate is related to reduced digestibility of the diet.

## Figures and Tables

**Table 1:** Mean chemical composition (g/kg DM) of substrates used in the gas in vitro incubations.

Item ^1^	Grass Silage	Barley
OM	940	972
CP	144	123
NDF	573	220

^1^ OM = organic matter; CP = crude protein; NDF = neutral detergent fiber assayed with heat-stable amylase and expressed exclusive of residual ash.

**Table 2:** Comparison of least square means for cow performance and gas production of the selected low and high CH_4_ emitter groups in vivo (*n* = 7 pairs of cows).

Item ^1^	Low	High	s.e.m. ^2^	*p*-Value ^3^
DMI kg/d	20.4	20.8	0.78	0.71
ECM kg/d	23.0	25.9	1.68	0.24
BW kg	596	644	30.4	0.28
CH_4_ g/d	331	442	19.6	<0.01
CH_4_/DMI kg/kg	16.7	21.5	130	0.02
Residual CH_4_ g/d	−44.5	46.1	18.0	<0.01

^1^ DMI = dry matter intake; ECM = energy corrected milk; BW = body weight; CH_4_ = total methane production; CH_4_/DMI = methane yield; Residual CH_4_ = calculated as the difference between observed and predicted total CH_4_ production according to linear regression model; ^2^ s.e.m = standard error of mean; and ^3^ *p*-value of the difference among groups (low vs. high emitters) according to a completely randomized design with the effect of pair of cows as a covariate in the model.

**Table 3:** Total volatile fatty acids (VFA) production, concentration, and molar proportion of each individual VFA of rumen fluid collected from both low and high emitters prior to the in vitro incubations (*n* = 7 runs/ pairs of cows).

Item ^1^	Low	High	s.e.m. ^2^	*p*-Value ^3^
Total VFA concentration (mmol/L)	46.7	50.4	4.65	0.60
VFA proportion (mmol/mol)				
Acetate	677	688	9.77	0.57
Propionate	156	158	4.92	0.78
Butyrate	132	138	9.24	0.65
Isobutyrate	10.7	10.9	0.64	0.83
Valerate	13.8	13.3	0.93	0.71
Isovalerate	5.47	4.94	0.42	0.40
Caproate	5.54	6.38	0.74	0.48
CH_4_VFA (moles/mol VFA)	379	377	3.97	0.78
pH	6.82	6.73	0.056	0.27

^1^ CH_4_VFA = Stoichiometry CH_4_ per mol of volatile fatty acids [23]; ^2^ s.e.m = standard error of mean; and ^3^ *p*-Value of the difference among groups (low vs. high emitters) according to a completely randomized design with the effect of pair of cows as a covariate in the model.

**Table 4:** The effects of rumen fluid inoculum (low vs. high emitters in vivo) on total gas production, predicted values of in vivo CH_4_ production, predicted CH_4_ production based on stoichiometric relationship with volatile fatty acids (VFA), and their kinetic parameters after 48-h incubation (*n* = 7 runs/ pairs of cows).

Item	In Vivo	Diet ^1^		CNSL ^2^		s.e.m. ^3^	*p*-Value			Interactions ^4^		
	Emitter	S	SB	Without	With		Emitter	Diet	CNSL	E × D	E × C	D × C
Asymptotic total gas prod. (mL/ g of DM)	Low	220	266	273	213	11.5	0.75	<0.01	<0.01	0.89	0.62	0.85
	High	221	268	272	217							
Predicted gas at 48 h	Low	158	218	214	162	7.40	0.70	<0.01	<0.01	0.95	0.61	0.55
(mL/ g of DM)	High	156	217	209	163							
Rate of total gas prod.	Low	0.047	0.075	0.064	0.058	0.0042	0.34	<0.01	0.01	0.73	0.79	0.48
(/h)	High	0.045	0.072	0.061	0.056							
Asymptotic CH_4_ prod.	Low	22.4	28.4	36.1	15.0	2.97	0.76	<0.01	<0.01	0.50	0.89	<0.01
(mL/ g of DM)	High	22.1	29.4	36.5	15.0							
Predicted in vivo CH_4_ at 48 h ^5^	Low	16.1	21.4	26.6	10.9	2.16	0.68	<0.01	<0.01	0.55	0.98	<0.01
(mL/ g DM)	High	16.0	22.1	26.9	11.3							
Rate of CH_4_ (/h)	Low	0.047	0.056	0.049	0.053	0.003	0.05	<0.01	0.01	0.95	0.03	<0.01
	High	0.050	0.058	0.049	0.058							
CH_4_ / total gas at 48 h	Low	0.099	0.107	0.126	0.080	0.0167	0.38	0.52	<0.01	0.81	0.26	0.94
	High	0.093	0.097	0.128	0.062							

^1^ S = 100% grass silage; SB = 50% grass silage + 50% barley. ^2^ CNSL (With) = 10 μL of CNSE dissolved in 450 μL of 99.5% ethanol. ^3^ s.e.m = standard error of mean. ^4^ E × D = Emitter × Diet; E × C = Emitter × CNSL; D × C = Diet × CNSL; E × D × C = Emitter × Diet × CNSL. ^5^ Methane was predicted in vivo by using a 50 h rumen retention time in the mechanistic rumen model by Ramin and Huhtanen [33].

**Table 5:** Total volatile fatty acids and production of each individual VFA after 48 h incubation from substrates incubated in rumen fluid from both low and high emitters in vivo in the gas in vitro system (*n* = 7 runs/ pairs of cows).

Item	In Vivo	Diet ^1^		CNSL ^2^		s.e.m ^3^	*p*-Value			Interactions ^4^		
	Emitter	S	SB	Without	With		Emitter	Diet	CNSL	E × D	E × C	D × C
Total VFA concentration ^4^	Low	67.5	63.0	65.8	64.7	4.14	0.26	0.13	<0.01	<0.01	0.01	0.14
(mmol/L)	High	56.4	68.4	69.2	55.7							
VFA prop. (mmol/mol)												
Acetate	Low	589	541	591	540	13.7	0.06	<0.01	<0.01	0.32	<0.01	0.03
	High	567	534	598	503							
Propionate	Low	266	293	245	314	16.4	<0.01	<0.01	<0.01	0.89	<0.01	<0.01
	High	286	315	246	355							
Butyrate	Low	85.6	109	102	92.1	4.74	0.09	<0.01	<0.01	0.04	0.94	0.09
	High	86.6	98.7	97.5	87.8							
Isobutyrate	Low	13.8	13.4	13.5	13.7	0.40	0.64	<0.01	0.03	0.16	0.13	0.01
	High	14.1	12.9	13.0	14.0							
Valerate	Low	25.2	26.0	27.2	24.0	2.24	0.10	0.16	0.01	0.02	0.33	0.99
	High	25.8	22.5	24.9	23.4							
Isovalerate	Low	11.0	11.1	11.4	10.7	0.54	0.86	<0.01	0.93	<0.01	0.01	0.01
	High	11.9	10.2	10.7	11.4							
Caproate	Low	9.05	6.87	10.7	5.18	2.39	0.37	0.02	<0.01	0.98	0.35	0.03
	High	8.23	6.09	9.11	5.21							
CH_4_VFA (moles/mol VFA) ^5^	Low	288	267	304	251	12.4	<0.01	<0.01	<0.01	0.99	<0.01	<0.01
	High	272	251	304	219							
pH	Low	6.33	6.42	6.36	6.46	0.021	0.02	<0.01	<0.01	0.51	0.84	0.46
	High	6.44	6.31	6.48	6.34							

^1^ S = 100% grass silage; SB = 50% grass silage + 50% barley. ^2^ CNSL (With) = 10 μL of CNSE dissolved in 450 μL of 99.5% ethanol. ^3^ s.e.m = standard error of mean. ^4^ E × D = Emitter × Diet; E × C = Emitter × CNSL; D × C = Diet × CNSL. ^4^ Total VFA concentrations and molar proportions are not corrected for blank. ^5^ CH_4_VFA = Stoichiometry CH_4_ per mole of volatile fatty acids [23].

## Data Availability

The data presented in this paper are available on request from the corresponding author.
